# P02-002 - IL36RN mutations in patients with DITRA

**DOI:** 10.1186/1546-0096-11-S1-A109

**Published:** 2013-11-08

**Authors:** JI Arostegui, MA Vicente-Villa, A Chaves, E Gonzalez-Roca, E Ruiz-Ortiz, J Rius, S Plaza, MA Gonzalez-Enseñat, J Yague

**Affiliations:** 1 Immunology, Hospital Clinic, Barcelona, Spain; 2 Dermatology, Hospital Sant Joan de Deu, Esplugues, Spain; 3 Dermatology, Hospital Infanta Cristina, Badajoz, Spain

## Introduction

Loss-of-function mutations in the *IL36RN* gene define a novel recessively inherited autoinflammatory syndrome named deficiency of IL-36 receptor antagonist (DITRA). This genetically determined deficiency was first described in a subgroup of patients with generalized pustular psoriasis. It is a life-threatening condition characterized by recurrent episodes of severe skin inflammation, with pustule development, associated with fever, malaise, extracutaneous involvement, neutrophilia and a marked acute phase response.

## Case report

### Methods

The patients’ data as well as the outcome of the administered treatments were collected from charts reviews. *IL36RN* analysis was performed by means of Sanger-based sequencing.

### Results

We describe two unrelated families with patients diagnosed as suffering from generalized pustular psoriasis. The first family is a large consanguineous Algerian family with several affected members living in Algeria and in Spain. The propositus is a 13 year-old child who had suffered from two episodes of severe skin inflammation, with disseminated pustular development and systemic features that required hospital admission. Once DITRA was described in 2011, this diagnosis was suggested for this patient. *IL36RN* mutational analyses revealed a homozygous T-to-C transition in the exon 3 (at c.80 position), which provokes the leucine-to-proline variant at residue 27 (p.L27P) of the protein. This missense variant has been previously identified as a true disease-causing mutation in other Maghrebian (Tunisian) families with DITRA.

The second family is an apparent non-consanguineous Spanish family with only one affected individual. The patient is a 15 year-old girl who suffered since 6 months of age from recurrent and severe episodes of generalized pustular psoriasis that required recurrent hospital admissions. She has been treated with different drugs, including methotrexate, acitretin, cyclosporin, phototherapy, etanercept, infliximab, adalimumab and ustekinumab, with variable and limited efficacy. As a DITRA diagnosis was suggested, *IL36RN* analysis was performed. This study revealed an apparent homozygous 7 bp deletion in the exon 5 (c.420_426del), which should provoke a frameshift of the normal open reading frame. Genetic studies are currently ongoing to elucidate the intrafamilial mutational segregation pattern.

## Discussion

We describe two novel families affected by the novel autoinflammatory disease called DITRA. The disease started in these patients during childhood as severe episodes of generalized pustular skin rash and systemic features that required hospital admissions. We identified a novel *IL36RN* mutation in the Spanish family, and the already known missense p.L27P mutation in the Algerian family. This insight probably expands the founder effect of this *IL36RN* mutation to other Maghrebian populations than Tunisian people.

## Disclosure of interest

None declared.
